# Supplementary material for: Promising Natural Compounds against Flavivirus Proteases: Citrus Flavonoids Hesperetin and Hesperidin
Source: Plants (Basel). 2021 Oct 14;10(10):2183. doi: 10.3390/plants10102183 (PMC8539695; doi:10.3390/plants10102183)
Supplement: Supplementary file 1 [file plants-10-02183-s001.zip › plants-1417391-supplementary.pdf]

Supplementary Information

Promising Natural Compounds against Flavivirus Proteases: Citrus Flavonoids Hesperetin and Hesperidin

Raphael J. Eberle[1,2*], Danilo S. Olivier[3], Marcos S. Amaral[4], Dieter Willbold[1,2,5], Raghuvir K. Arni[6], Monika A. Coronado[1*]

[1]Institute of Biological Information Processing (IBI-7: Structural Biochemistry), Forschungszentrum Jülich, Jülich, Germany.

[2]Institut für Physikalische Biologie, Heinrich-Heine-Universität Düsseldorf, Universitätsstraße, Düsseldorf, Germany.

[3]Federal University of Tocantins, Araguaína-TO, Brazil.

[4]Institute of Physics, Federal University of Mato Grosso do Sul, Campo Grande-MS, Brazil.

[5]JuStruct: Jülich Centre for Structural Biology, Forchungszentrum Jülich, Jülich, Germany.

[6]Multiuser Center for Biomolecular Innovation, Departament of Physics, Instituto de Biociências Letras e Ciências Exatas (Ibilce), Universidade Estadual Paulista (UNESP), São Jose do Rio Preto-SP, Brazil.

[#]both authors contributed equally

*Correspondence to r.eberle@fz-juelich.de or m.coronado@fz-juelich.de

Table of contents

Supplementary Figure S1. SDS Gels after expression and purification of DENV2, YFV and WNV NS2B/NS3^{pro}.
Supplementary Figure S2. HST and HST inhibitory activity against DENV2, YFV and WNV NS2B/NS3^{pro}.
Supplementary Figure S3. Fluorescence spectroscopy of Trp at 295 nm of DENV2, YFV and WNV NS2B/NS3^{pro} in the presence of HST.
Supplementary Figure S4. Fluorescence spectroscopy of Trp at 295 nm of DENV2, YFV and WNV NS2B/NS3^{pro} in the presence of HSD.
Supplementary Figure S5. Time dependent modifications of the DENV2 NS2B/NS3^{pro} structure.
Supplementary Figure S6. Time dependent modifications of the YFV NS2B/NS3^{pro} structure.
Supplementary Figure S7. Time dependent modifications of the WNV NS2B/NS3^{pro} structure.
Supplementary Figure S8. Time dependent modifications of the DENV2 NS2B/NS3^{pro}-HST complex.
Supplementary Figure S9. Time dependent modifications of the YFV NS2B/NS3^{pro}-HST complex.
Supplementary Figure S10. Time dependent modifications of the WNV NS2B/NS3^{pro}-HST complex.
Supplementary Figure S11. Time dependent modifications of the DENV2 NS2B/NS3^{pro}-HSD complex.
Supplementary Figure S12. Time dependent modifications of the YFV NS2B/NS3^{pro}-HSD complex.
Supplementary Figure S13. Time dependent modifications of the WNV NS2B/NS3^{pro}-HSD complex.
Supplementary Figure S14. 3D representation of NS2B/NS3^{pro}-HST and –HSD complex.
Supplementary Figure S15. Decomposition of the binding energy of DENV2 NS2B/NS3^{pro}-HST complex of two independent replica.
Supplementary Figure S16. Decomposition of the binding energy of YFV NS2B/NS3^{pro}-HST complex of two independent replica.
Supplementary Figure S17. Decomposition of the binding energy of WNV NS2B/NS3^{pro}-HST complex of two independent replica.
Supplementary Figure S18. Decomposition of the binding energy of DENV2 NS2B/NS3^{pro}-HSD complex of two independent replica.
Supplementary Figure S19. Decomposition of the binding energy of YFV NS2B/NS3^{pro}-HSD complex of two independent replica.
Supplementary Figure S20. Decomposition of the binding energy of WNV NS2B/NS3^{pro}-HSD complex of two independent replica.
Supplementary Text 1. Expression and purification of NS2B/NS3 proteases.

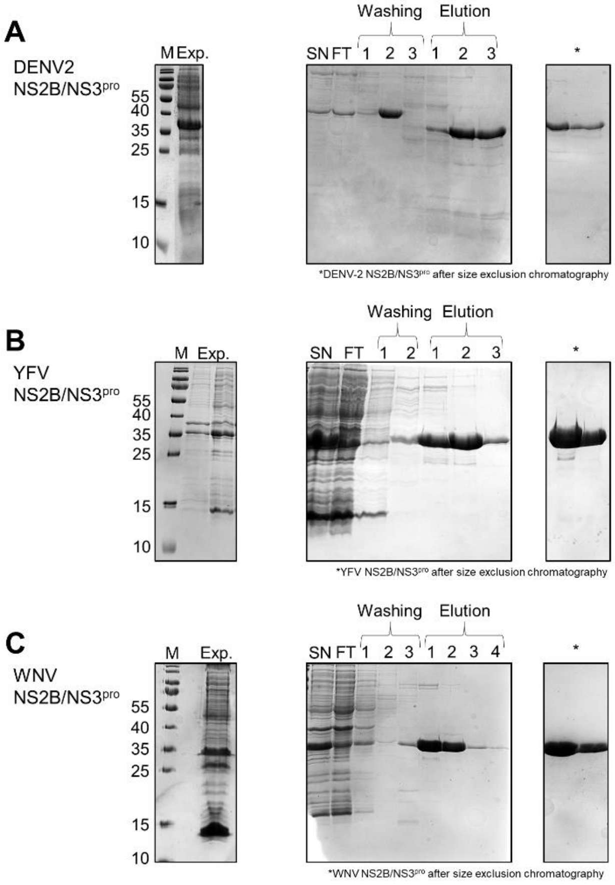

Figure S1. SDS Gels after expression and purification of DENV2, YFV and WNV NS2B/NS3^{pro}. SDS 15% Gels of NS2B/NS3^{pro} purification with Ni-NTA sepharose. M: protein marker, Exp.: cells after expression time, SN: supernatant, FT: flow through, Washing: washing steps, Elution: elution steps with imidazole. The SDS 15% Gel demonstrate the purity of the proteases after size exclusion chromatography. **(A)** DENV2 NS2B/NS3^{pro}, **(B)** YFV NS2B/NS3^{pro} and **(C)** WNV NS2B/NS3^{pro}.

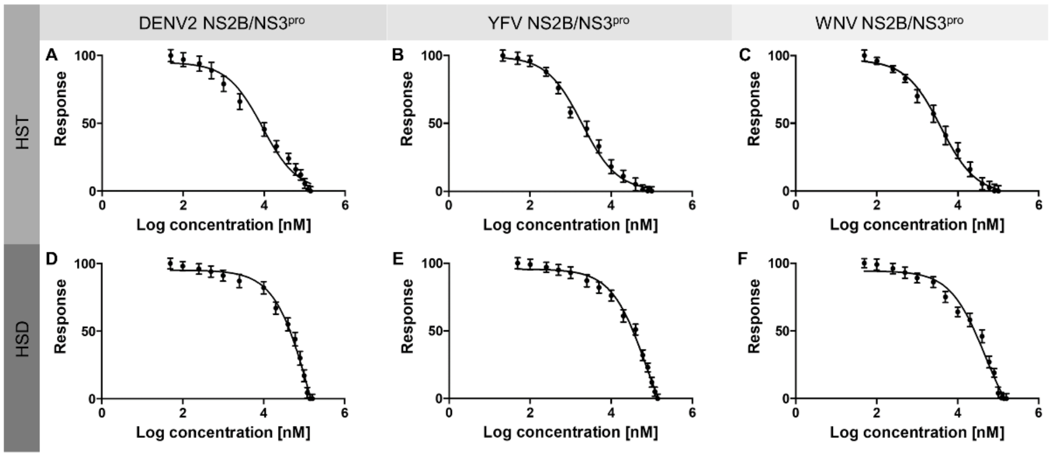

Figure S2. HST and HST inhibitory activity against DENV2, YFV and WNV NS2B/NS3^{pro}. Dose response curves for IC_{50} determination. The normalized response [%] of flavivirus proteases are plotted against the Log of the HST and HSD concentration. **(A)** Dose response curve of HST and DENV2 NS2B/NS3^{pro}. **(B)** Dose response curve of HST and YFV NS2B/NS3^{pro}. **(C)** Dose response curve of HST and WNV NS2B/NS3^{pro}. **(D)** Dose response curve of HSD and DENV2 NS2B/NS3^{pro}. **(E)** Dose response

curve of HSD and YFV NS2B/NS3^{pro}. **(F)** Dose response curve of HSD and WNV NS2B/NS3^{pro}. Data shown are the mean ± SD from three independent measurements (n=3).

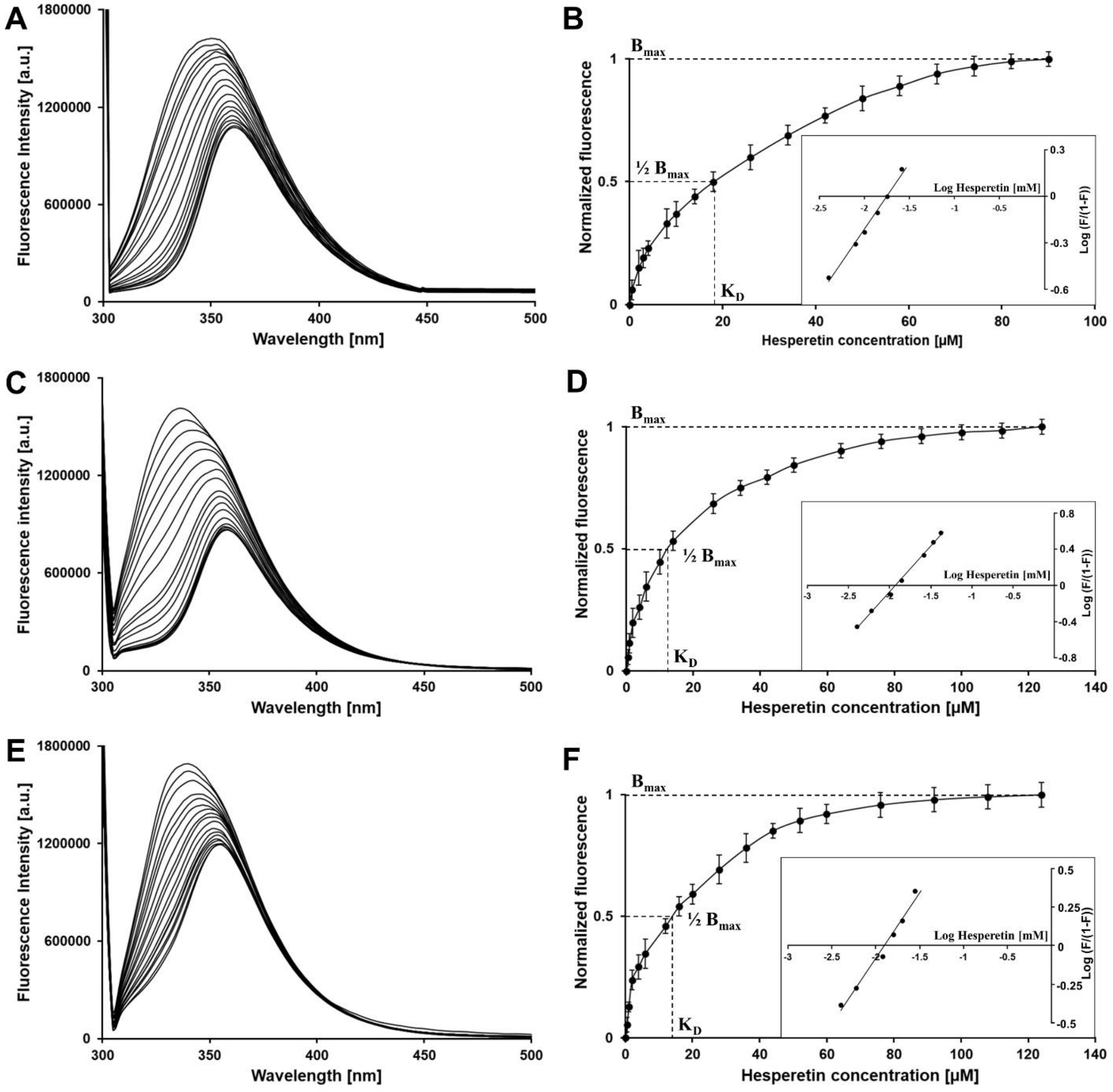

Figure S3. Fluorescence spectroscopy of Trp at 295 nm of DENV2, YFV and WNV NS2B/NS3^{pro} in the presence of HST. **(A)** Fluorescence of DENV2 NS2B/NS3^{pro} under influence of HST titration. **(B)** Binding saturation curve determined a K_D value of 18.7 ± 2.7 µM for the DENV2 NS2B/NS3^{pro}-HST interaction. K_D determination using a modified Hill equation, intersection with x-axis corresponds to the logarithmic value of the K_D. **(C)** Fluorescence of YFV NS2B/NS3^{pro} under influence of HST titration. **(D)** Binding saturation curve determined a K_D value of 12.3 ± 2.2 µM for the YFV NS2B/NS3^{pro}-HST interaction. K_D determination using a modified Hill equation, intersection with x-axis corresponds to the logarithmic value of the K_D. **(E)** Fluorescence of WNV NS2B/NS3^{pro} under influence of HST titration. **(D)** Binding saturation curve determined a K_D value of 13.5 ± 2.0 µM for the WNV NS2B/NS3^{pro}-HST interaction. K_D determination using a modified Hill equation, intersection with x-axis corresponds to the logarithmic value of the K_D. Data shown are the mean ± SD from three independent measurements (n=3).

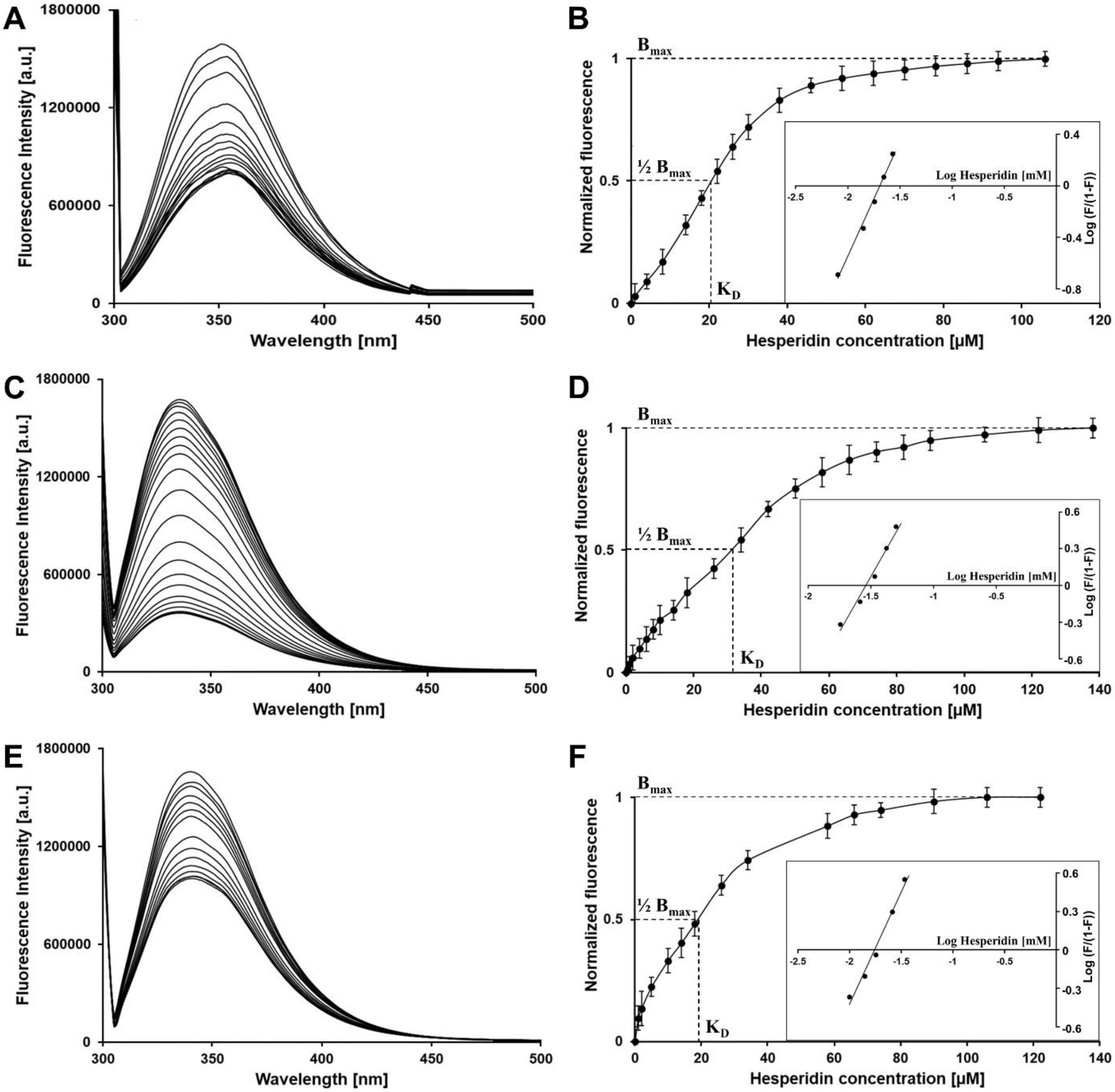

Figure S4. Fluorescence spectroscopy of Trp at 295 nm of DENV2, YFV and WNV NS2B/NS3^{pro} in the presence of HSD. **(A)** Fluorescence of DENV2 NS2B/NS3^{pro} under influence of HSD titration. **(B)** Binding saturation curve determined a K_D value of 20.9 ± 2.4 µM for the DENV2 NS2B/NS3^{pro}-HSD interaction. K_D determination using a modified Hill equation, intersection with x-axis corresponds to the logarithmic value of the K_D. **(C)** Fluorescence of YFV NS2B/NS3^{pro} under influence of HSD titration. **(D)** Binding saturation curve determined a K_D value of 29.5 ± 2.8 µM for the YFV NS2B/NS3^{pro}-HSD interaction. K_D determination using a modified Hill equation, intersection with x-axis corresponds to the logarithmic value of the K_D. **(E)** Fluorescence of WNV NS2B/NS3^{pro} under influence of HSD titration. **(D)** Binding saturation curve determined a K_D value of 19.5 ± 2.8 for the WNV NS2B/NS3^{pro}-HSD interaction. K_D determination using a modified Hill equation, intersection with x-axis corresponds to the logarithmic value of the K_D. Data shown are the mean ± SD from three independent measurements (n=3).

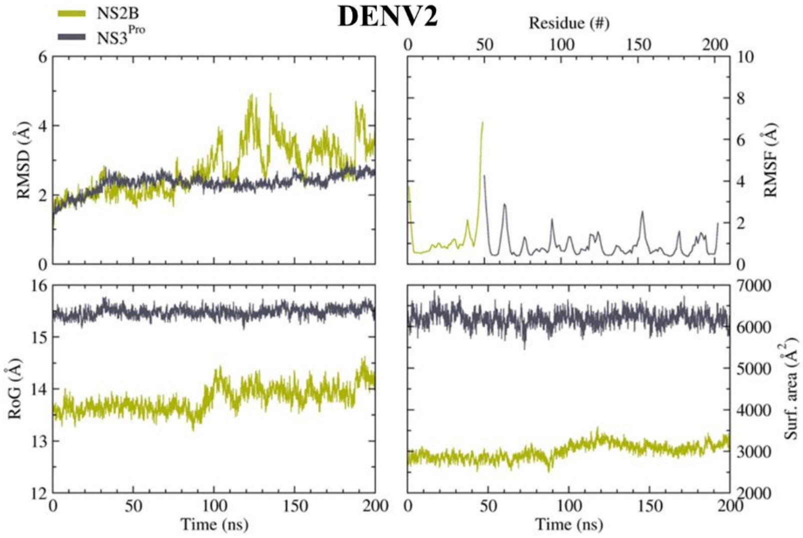

Figure S5. Time dependent modifications of the DENV2 NS2B/NS3^{pro} structure. NS2B (grey) and NS3^{pro} (yellow). RMSD, RMSF, RoG and surface area as function of time. RMSF for each amino acid.

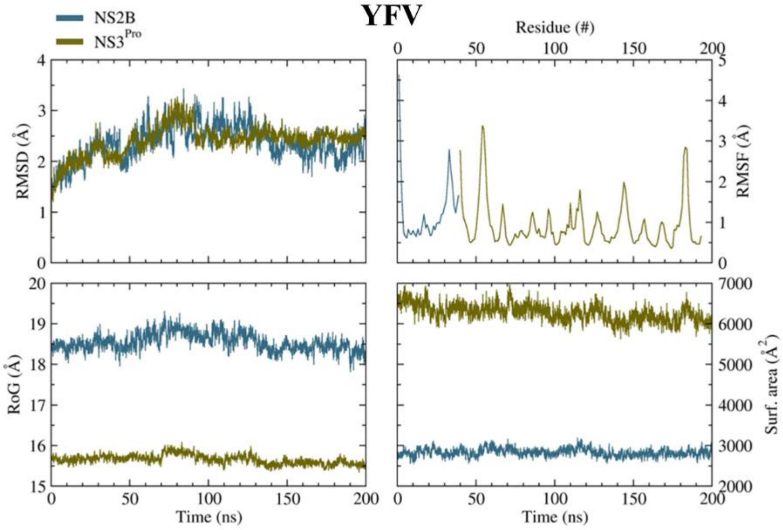

Figure S6. Time dependent modifications of the YFV NS2B/NS3^{pro} structure. NS2B (blue) and NS3^{pro} (green). RMSD, RMSF, RoG and surface area as function of time. RMSF for each amino acid.

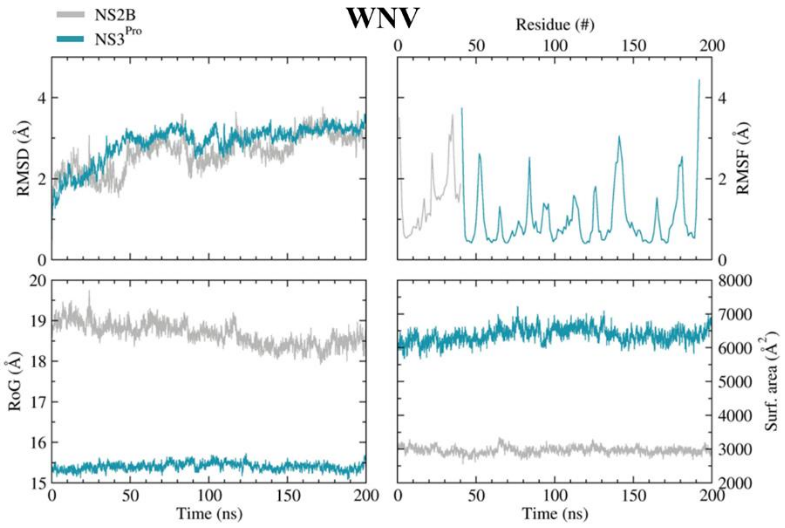

Figure S7. Time dependent modifications of the WNV NS2B/NS3^{pro} structure. NS2B (light grey) and NS3^{pro} (blue). RMSD, RMSF, RoG and surface area as function of time. RMSF for each amino acid.

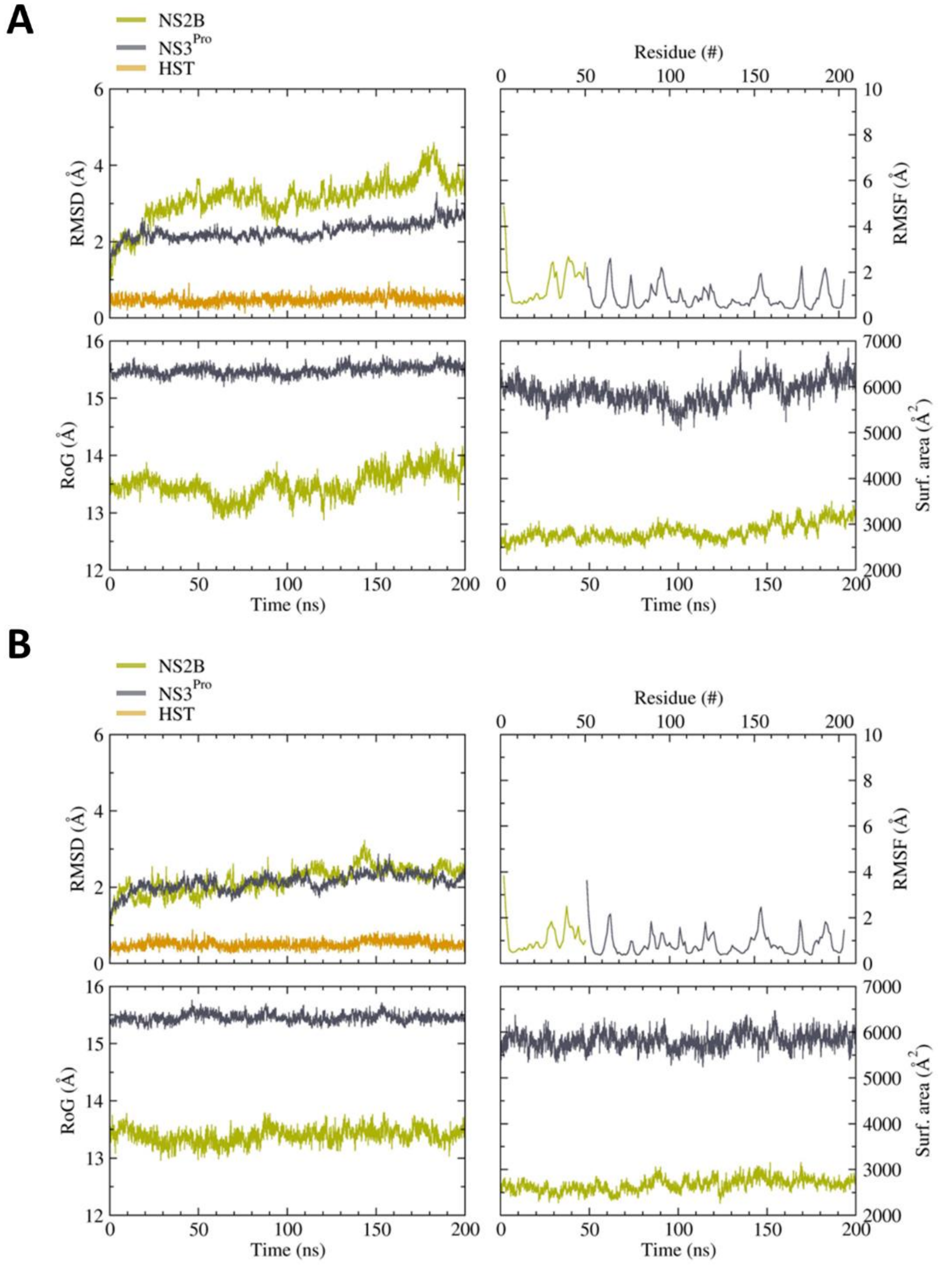

Figure S8. Time dependent modifications of the DENV2 NS2B/NS3^{pro}-HST complex. NS2B (green), NS3^{pro} (grey) and HST (yellow). RMSD, RMSF, RoG and surface area as function of time. RMSF for each amino acid. **(A)** replicate one and **(B)** replicate two.

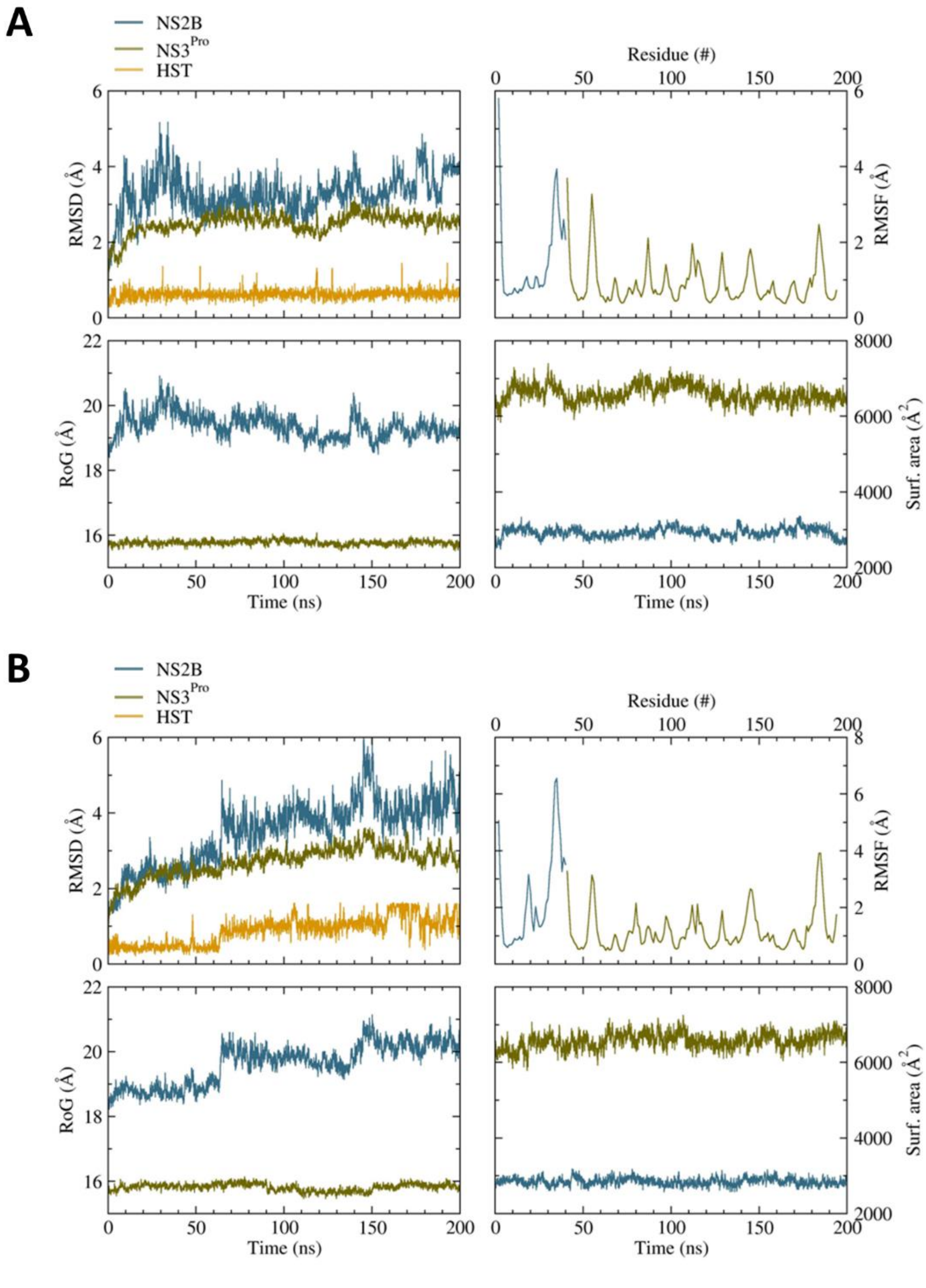

Figure S9. Time dependent modifications of the YFV NS2B/NS3^{pro}-HST complex. NS2B (purple), NS3^{pro} (brown) and HST (yellow). RMSD, RMSF, RoG and surface area as function of time. RMSF for each amino acid. **(A)** replicate one and **(B)** replicate two.

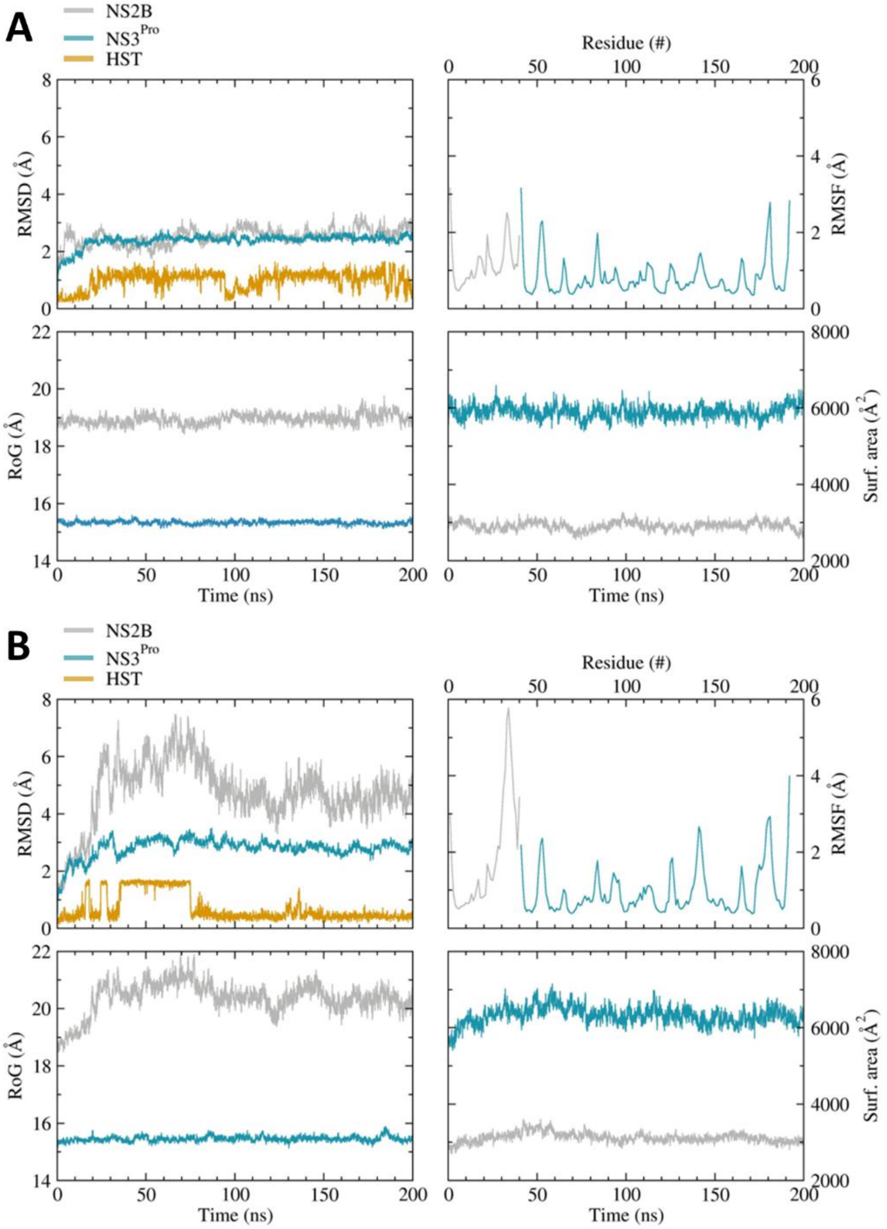

Figure S10. Time dependent modifications of the WNV NS2B/NS3pro-HST complex. NS2B (grey), NS3pro (blue) and HST (yellow). RMSD, RMSF, RoG and surface area as function of time. RMSF for each amino acid. **(A)** replicate one and **(B)** replicate two.

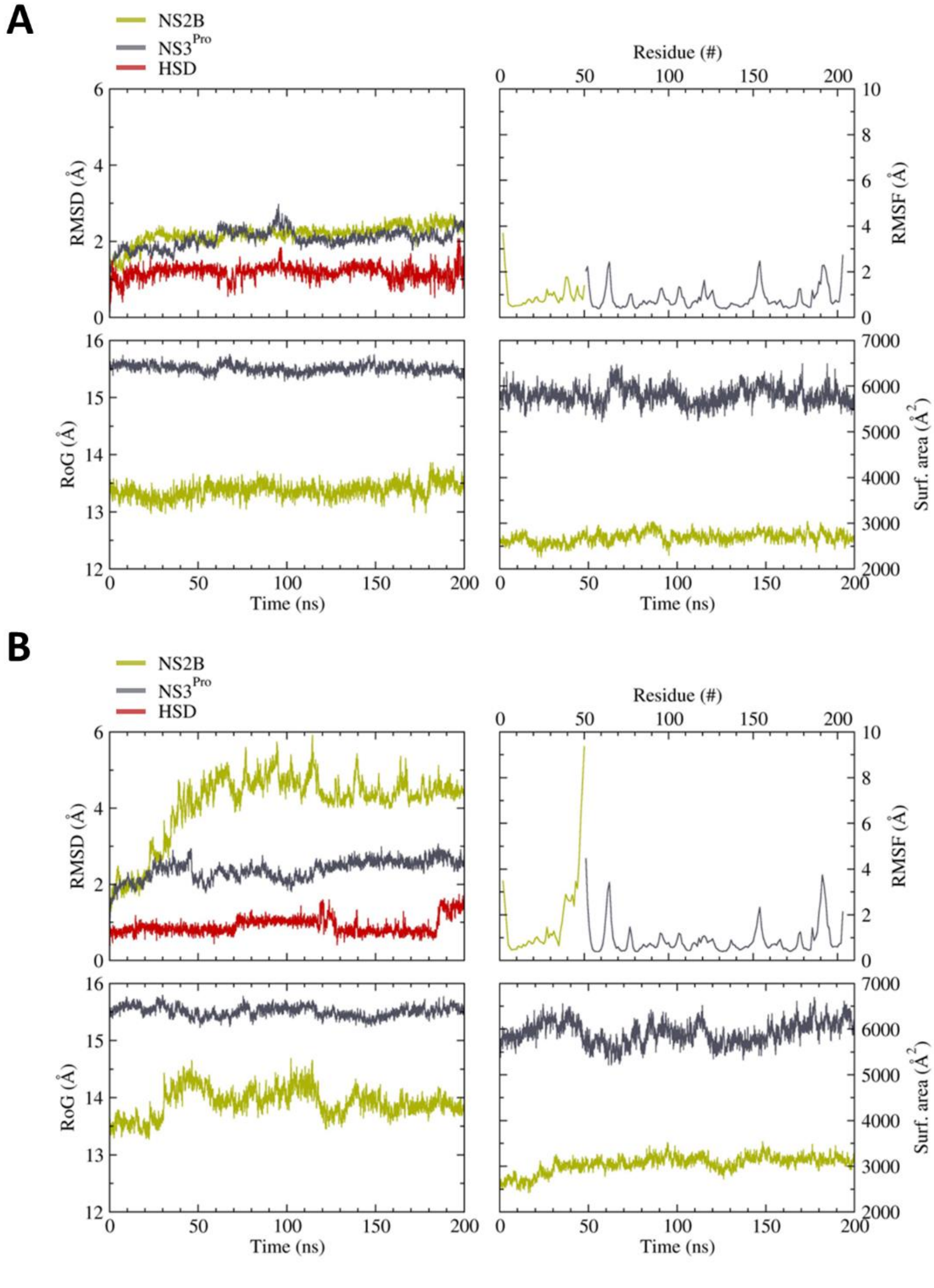

Figure S11. Time dependent modifications of the DENV2 NS2B/NS3^{pro}-HSD complex. NS2B (green), NS3^{pro} (grey) and HSD (red). RMSD, RMSF, RoG and surface area as function of time. RMSF for each amino acid. **(A)** replicate one and **(B)** replicate two.

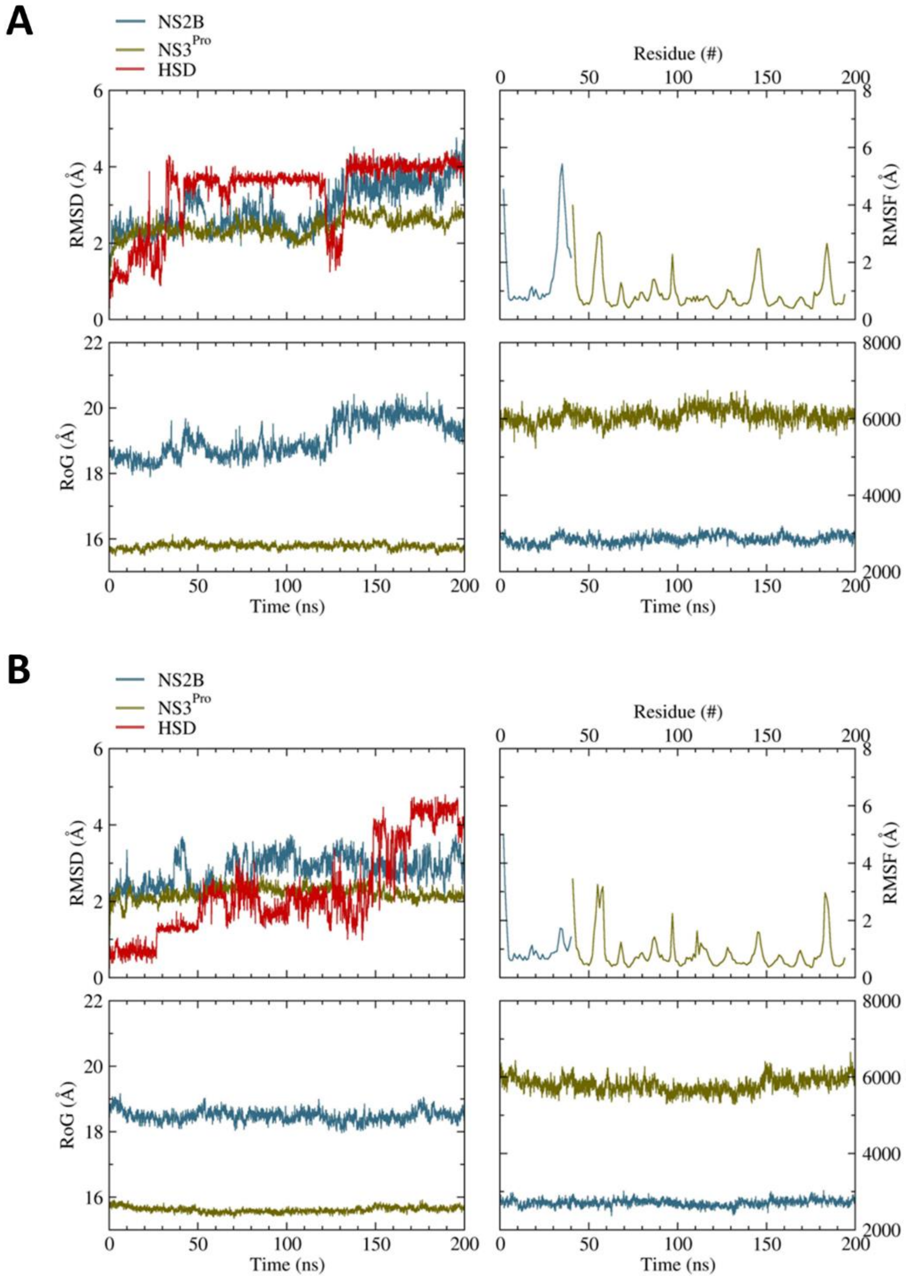

Figure S12. Time dependent modifications of the YFV NS2B/NS3^{pro}-HSD complex. NS2B (purple), NS3^{pro} (browny) and HSD (red). RMSD, RMSF, RoG and surface area as function of time. RMSF for each amino acid. **(A)** replicate one and **(B)** replicate two.

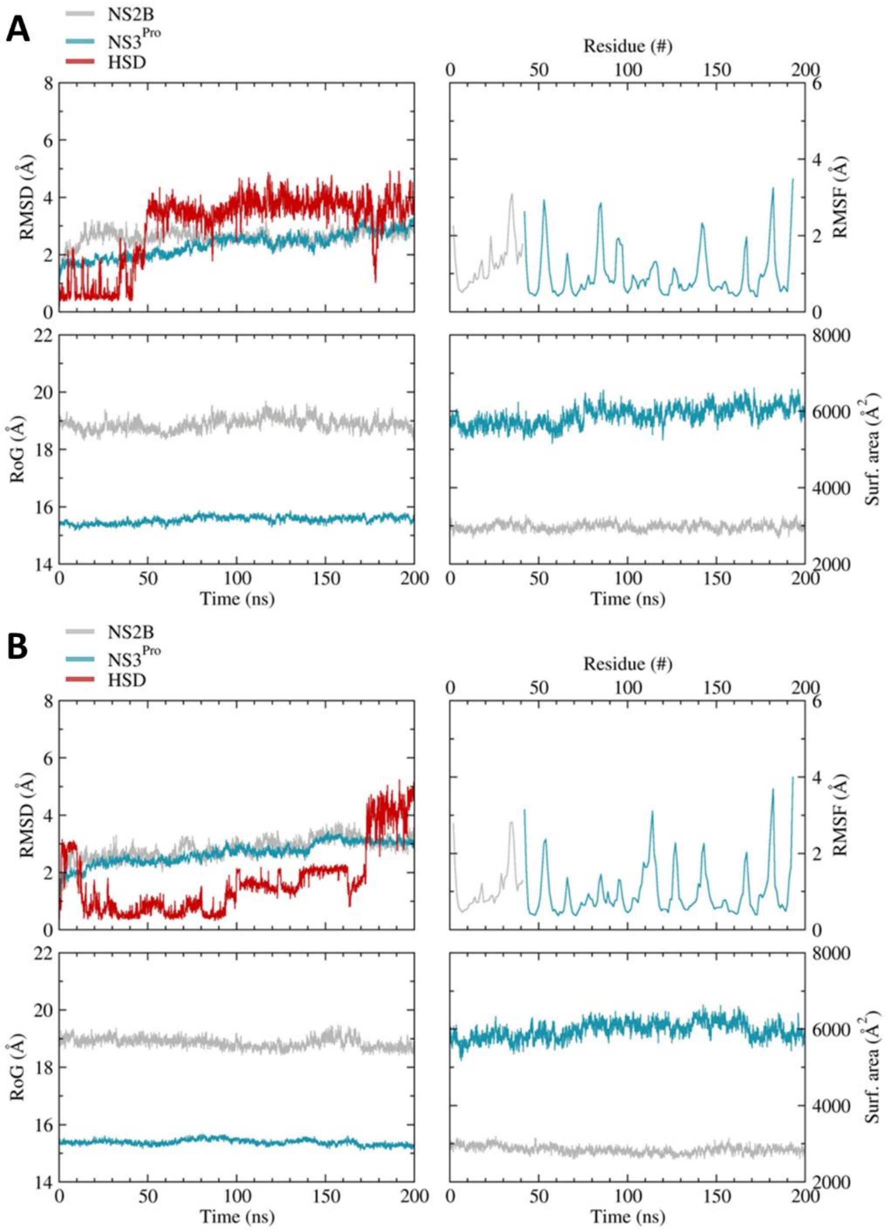

Figure S13. Time dependent modifications of the WNV NS2B/NS3^{pro}-HSD complex. NS2B (grey), NS3^{pro} (blue) and HSD (red). RMSD, RMSF, RoG and surface area as function of time. RMSF for each amino acid. **(A)** replicate one and **(B)** replicate two.

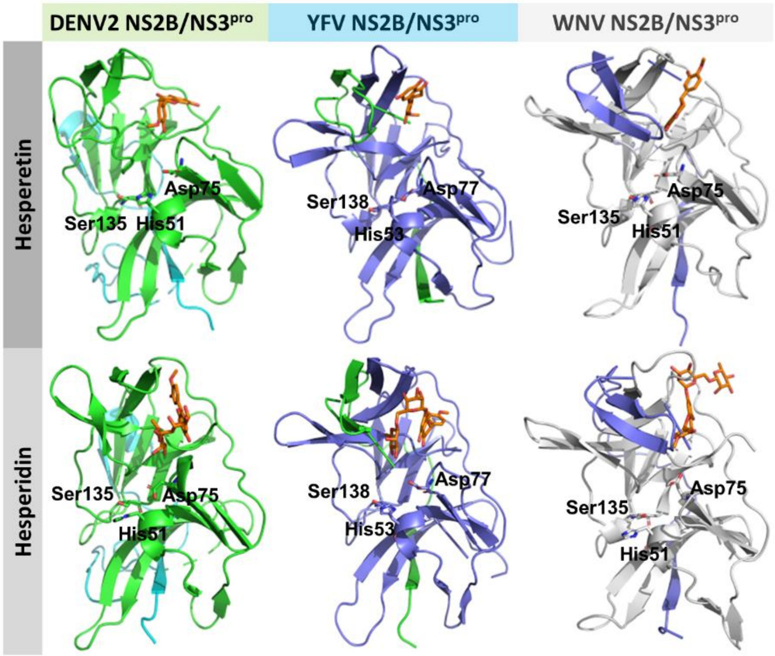

Figure S14. 3D representation of NS2B/NS3^{pro}-HST and –HSD complex (NS2B/NS3^{pro} in ribbon presentation, active site triad, HST and HSD in sticks). NS2B (DENV2: turquoise, YFV: green, WNV: blue) and NS3pro (DENV2: green, YFV: blue, WNV: light grey) are colored differently, HST and HSD in orange.

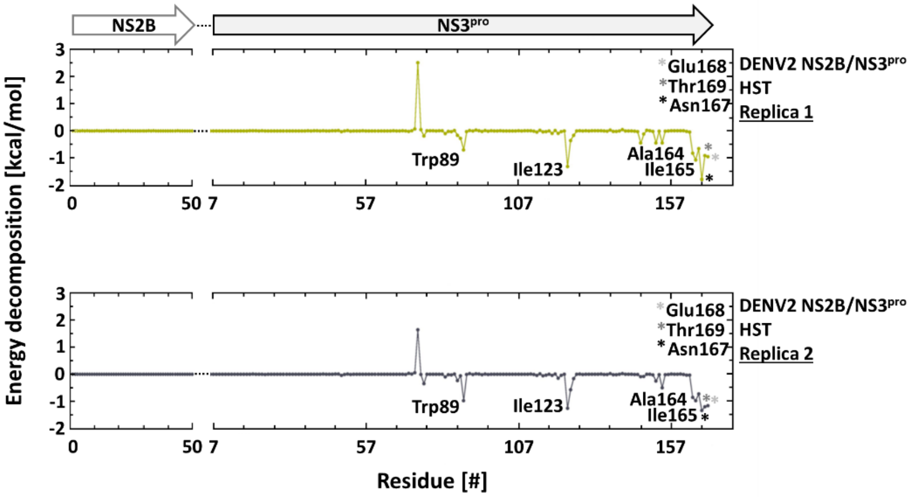

Figure S15. Decomposition of the binding energy of DENV2 NS2B/NS3^{pro}-HST complex of two independent replica. NS2B and NS3^{pro} are labelled and by arrows. The amino acid residues involved in the interaction with HST are labeled with name and sequence number.

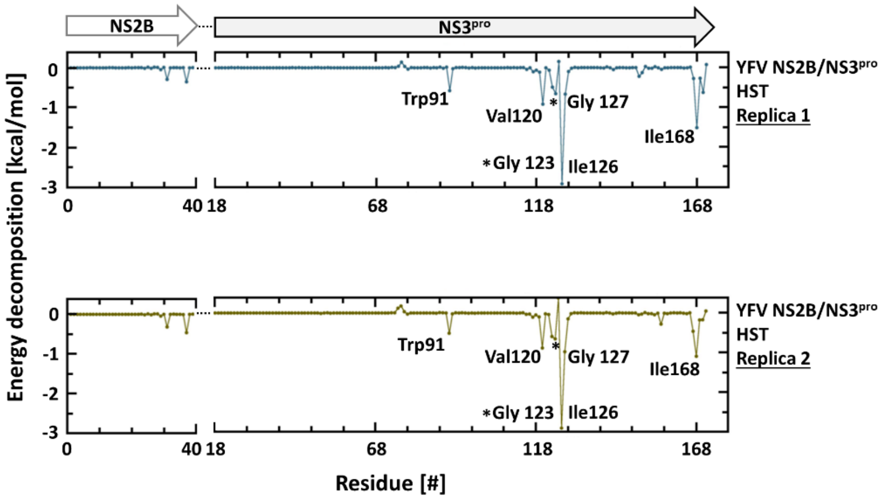

Figure S16. Decomposition of the binding energy of YFV NS2B/NS3^{pro}-HST complex of two independent replica. NS2B and NS3^{pro} are labelled and by arrows. The amino acid residues involved in the interaction with HST are labeled with name and sequence number.

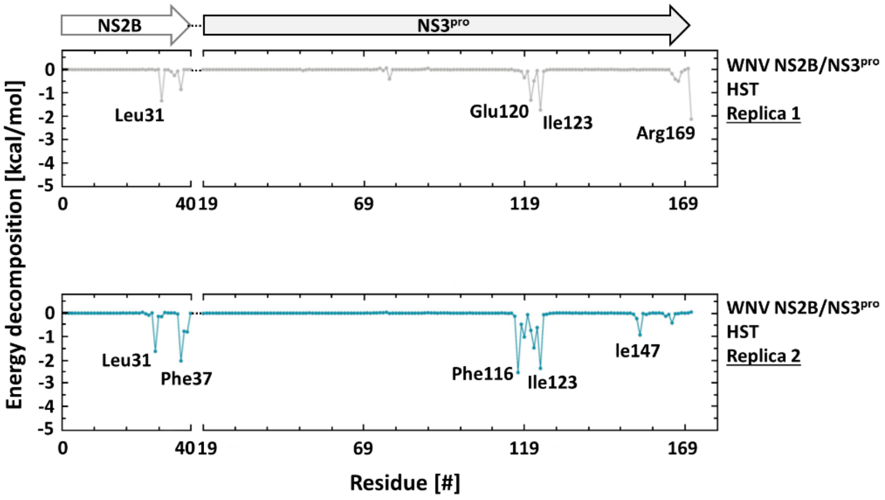

Figure S17. Decomposition of the binding energy of WNV NS2B/NS3^{pro}-HST complex of two independent replica. NS2B and NS3^{pro} are labelled and by arrows. The amino acid residues involved in the interaction with HST are labeled with name and sequence number.

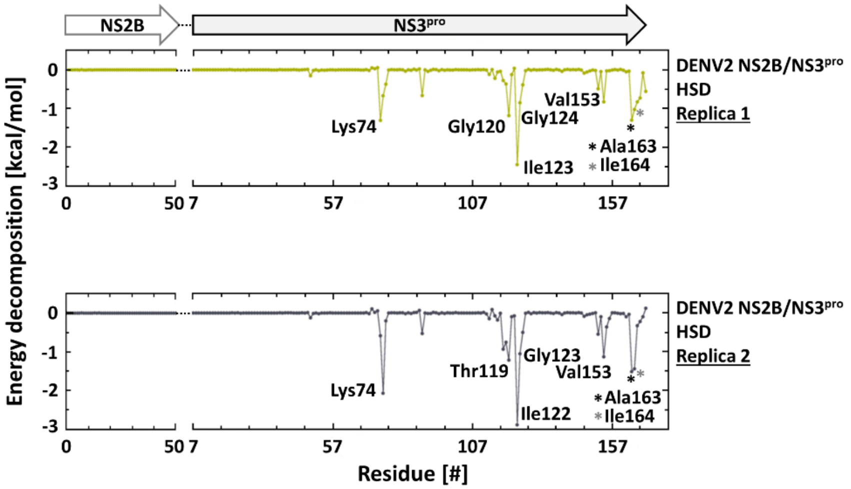

Figure S18. Decomposition of the binding energy of DENV2 NS2B/NS3^{pro}-HSD complex of two independent replica. NS2B and NS3^{pro} are labelled and by arrows. The amino acid residues involved in the interaction with HST are labeled with name and sequence number.

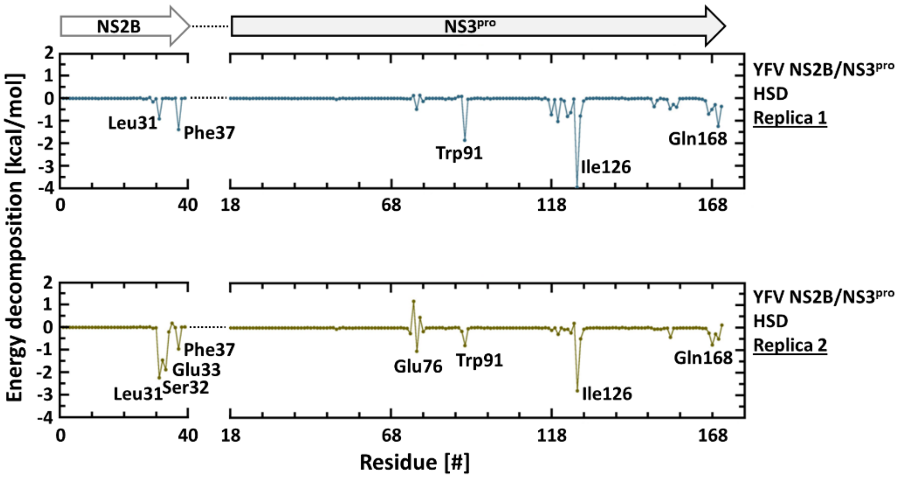

Figure S19. Decomposition of the binding energy of YFV NS2B/NS3^{pro}-HSD complex of two independent replica. NS2B and NS3^{pro} are labelled and by arrows. The amino acid residues involved in the interaction with HST are labeled with name and sequence number.

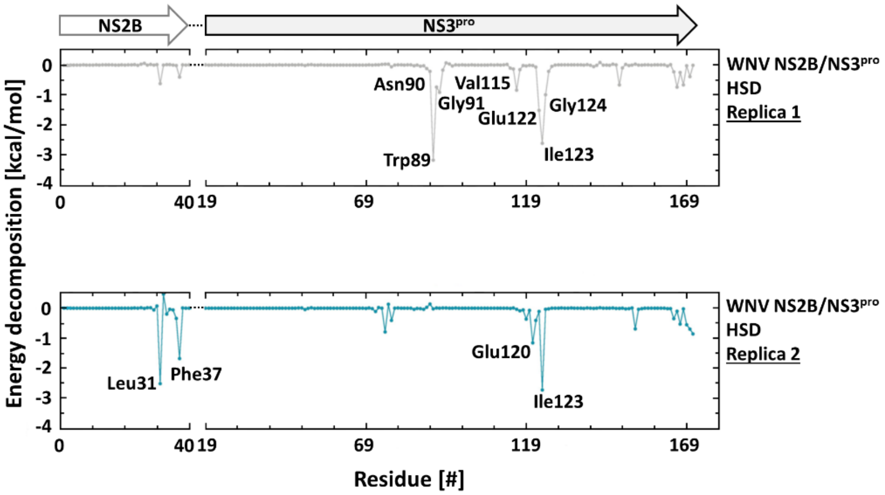

Figure S20. Decomposition of the binding energy of WNV NS2B/NS3pro-HSD complex of two independent replica. NS2B and NS3pro are labelled and by arrows. The amino acid residues involved in the interaction with HST are labeled with name and sequence number.

Supplementary Text 1. Expression and purification of NS2B/NS3 proteases.

DENV2 NS2B/NS3pro

DENV2 NS2B/NS3pro-pET15 vectors were transformed into *E. coli* Lemo (DE3) (New England Biolabs, USA) competent cells, and grown overnight at 37ºC in LB-medium. This pre-culture was added to fresh 2xYT-medium (Antibiotics: ampicillin and chloramphenicol) and grew at 37ºC until the cells reached OD_{600} of 0.6-0.8. The culture was induced with final concentration of 0.5 mM IPTG and incubated for 12 hours, at 16ºC and 100 rpm. Later, the culture was harvested by centrifugation at 4,000 rpm, 5 °C for 20 min, discarding the supernatant and re-suspending the DENV2 NS2B/NS3pro cell pellet in 20 mM Bis-Tris-HCL pH 6.3, 500 mM NaCl and protease inhibitor (Roche, Basel, Switzerland). Then, the cell-suspension was incubated on ice for 1 h with lysozyme, subsequently being lysed by sonication in four sets of 30 s pulses of 30% amplitude, with 10 s intervals. This method obtained crude cell extract, which was centrifuged at 8,000 rpm, 6 °C for 90 min. The supernatant containing DENV2 NS2B/NS3pro was loaded onto Ni-NTA column pre-equilibrated with 20 mM Bis-Tris-HCL pH 6.3, 500 mM NaCl extensively washed

with the same buffer, containing 0, 20 and 50 mM imidazole the protein eluted stepwise at 100, 250 and 500 mM imidazole. The eluted fractions were concentrated and injected onto a Superdex 75 HR 10/30 size exclusion column (GE Healthcare, USA), pre-equilibrated with 20 mM Tris-HCl, pH 8.0, 300 mM NaCl, 5 % (v/v) glycerol. Sample purity after each purification step was assessed by 15 % SDS-PAGE.

YFV NS2B/NS3^{pro}

YFV NS2B/NS3^{pro}-pET-24a (+) vectors were transformed into *E. coli* Lemo (DE3) (New England BioLabs, USA) competent cells, and grown overnight at 37 ºC in LB-medium. This pre-culture was added to fresh LB-medium (Antibiotics: kanamycin and chloramphenicol) and grew at 37 ºC until the cells reached an OD_{600} of 0.8. Gene expression was induced with final concentration of 1.0 mM IPTG and incubated overnight, at 18 ºC and 130 rpm. Subsequently, the culture was harvested by centrifugation at 4,000 rpm at 5 °C for 20 min (Sorvall RC-5B Plus Superspeed Centrifuge, Thermo Fisher Scientific, USA; GSA rotor) the supernatant was discarded and the cells containing recombinant YFV NS2B/NS3^{pro} were resuspended in 50 mM Tris-HCl pH 7.5, 500 mM NaCl, 1mM TCEP, 10% Glycerol. The cell-suspension was incubated on ice for 1 h with lysozyme and was subsequently lysed by sonication in four pulses of 30 s each, and an amplitude of 30% interspersed with intervals of 10 s and the crude cell extract obtained was centrifuged at 8,000 rpm and 6 °C for 90 min (Sorvall RC-5B Plus Superspeed Centrifuge, Thermo Fisher Scientific, GSA rotor). The supernatant containing YFV NS2B/NS3^{pro} was loaded onto a Ni-NTA column pre-equilibrated with 50 mM Tris-HCl pH 7.5, 500 mM NaCl, 1mM TCEP, 10% Glycerol extensively washed with the same buffer containing 0 and 20 mM imidazole, NS2B/NS3^{pro} eluted stepwise with 100, 200 and 500 mM imidazole. The eluted fractions were concentrated and injected onto a Superdex 75 10/30 GL size exclusion chromatography (GE

Healthcare, USA), pre-equilibrated with 20 mM Tris-HCl, pH 7.5, 150 mM NaCl, 1 mM TCEP, 4% Glycerol. Sample purity after each purification step was assessed by 15% SDS-PAGE gels.

WNV NS2B/NS3^{pro}

WNV NS2B/NS3^{pro}-pET-15b vectors were transformed into *E. coli* Lemo (DE3) (New England BioLabs, USA) competent cells, and grown overnight at 37 ºC in LB-medium. This pre-culture was added to fresh 2xYT-medium (Antibiotics: ampicillin and chloramphenicol) and grew at 37 ºC until the cells reached an OD_{600} of 0.8. Gene expression was induced with final concentration of 0.5 mM IPTG and incubated overnight, at 25 ºC and 130 rpm. Subsequently, the culture was harvested by centrifugation at 4,000 rpm at 5 °C for 20 min (Sorvall RC-5B Plus Superspeed Centrifuge, Thermo Fisher Scientific, USA; GSA rotor) the supernatant was discarded and the cells containing recombinant WNV NS2B/NS3^{pro} were resuspended in 25 mM Tris-HCl pH 8.5, 500 mM NaCl, 10% Glycerol. The cell-suspension was incubated on ice for 1 h with lysozyme and was subsequently lysed by sonication in four pulses of 30 s each, and an amplitude of 30% interspersed with intervals of 10 s and the crude cell extract was centrifuged at 8,000 rpm and 6 °C for 90 min (Sorvall RC-5B Plus Superspeed Centrifuge, Thermo Fisher Scientific, GSA rotor). The supernatant containing WNV NS2B/NS3^{pro} was loaded onto a Ni-NTA column pre-equilibrated with 25 mM Tris-HCl pH 8.5, 500 mM NaCl, 10% Glycerol, extensively washed with the same buffer containing 0, 10 and 20 mM imidazole, WNV NS2B/NS3^{pro} eluted stepwise with 75, 100, 150 and 500 mM imidazole. The eluted fractions were concentrated and injected onto a Superdex 75 10/300 GL size exclusion column (GE Healthcare, USA), pre-equilibrated with 25 mM Tris-HCl, pH 8.5, 150 mM NaCl, 5% Glycerol. Sample purity after each purification step was assessed by 15% SDS-PAGE gels.
